# Efficient full Monte Carlo modelling and multi-energy generative model development of an advanced X-ray device

**DOI:** 10.1016/j.zemedi.2022.04.006

**Published:** 2022-06-07

**Authors:** Hermann Fuchs, Lukas Zimmermann, Niklas Reisz, Markus Zeilinger, Alexander Ableitinger, Dietmar Georg, Peter Kuess

**Affiliations:** a Medical University of Vienna, Department of Radiation Oncology, Währinger Gürtel 18–20, 1090 Wien, Austria; b MedAustron Ion Therapy Center, Marie-Curie-Straße 5, 2700 Wiener Neustadt, Austria; c Faculty of Health, University of Applied Sciences Wiener Neustadt, Johannes-Gutenberg-Straße 3, Wiener Neustadt, Austria; d Competence Center for Preclinical Imaging and Biomedical Engineering, University of Applied Sciences Wiener Neustadt, Johannes-Gutenberg-Straße 3, 2700 Wiener Neustadt, Austria; e Complexity Science Hub Vienna, Josefstädter Strasse 39, 1080 Vienna, Austria

**Keywords:** X-ray, Monte Carlo, Neuronal networks, Machine learning

## Abstract

Monte Carlo (MC) simulations of X-ray image devices require splitting the simulation into two parts (*i.e.* the generation of x-rays and the actual imaging). The X-ray production remains unchanged for repeated imaging and can thus be stored in phase space (PhS) files and used for subsequent MC simulations. Especially for medical images these dedicated PhS files require a large amount of data storage, which is partly why Generative Adversarial Networks (GANs) were recently introduced. We enhanced the approach by a conditional GAN to model multiple energies using one network. This study compares the use of PhSs, GANs, and conditional GANs as photon source with measurements.

An X-ray -based imaging system (*i.e.* ImagingRing) was modelled in this study. half-value layers (HVLs), focal spot, and Heel effect were measured for subsequent comparison. MC simulations were performed with GATE-RTion v 1.0 considering the geometry and materials of the imaging system with vendor specific schematics. A traditional GAN model as well as the favourable conditional GAN was implemented for PhS generation.

Results of the MC simulation were in agreement with the measurements regarding HVL, focal spot, and Heel effect. The conditional GAN performed best with a non-saturated loss function with R1 regularisation and gave similarly results as the traditional GAN approach.

GANs proved to be superior to the PhS approach in terms of data storage and calculation overhead. Moreover, a conditional GAN enabled an energy interpolation to separate the network training process from the final required X-ray energies.

## 1 Introduction

Monte Carlo (MC) methods can provide a description of a system obtained by stochastic experiments. In medical physics, MC simulations are extensively used for many years [1] with countless applications ranging from dosimetry, nuclear medicine or imaging [2], [3], [4]. Especially for X-ray imaging accurate simulations are beneficial to account for scattering components and to fine tune the imaging quality and reduce the imaging dose [3]. For very high quality models, the whole mechanical structures need to be modelled and the path of the particles traversing the full geometry is simulated *e.g.* starting from the X-ray source to the detector response.

Unfortunately, simulating an X-ray device starting with the initial electron beam is calculation inefficient. A straightforward approach is to split the simulation into two parts. In the first part, the X-ray beam production is simulated, *i.e.* the primary electron beam, its transport to the target and its subsequent conversion to x-rays as well as static beam shaping elements.

In the second part, the actual imaging is simulated including the effect of modular beam shaping elements, such as collimators, as well as the object projection. The first part remains unchanged for repeated imaging, consequently produces the same output and can be pre-calculated. This output can be stored in so-called phase space (PhS) files, which can be understood as a dictionary for photons or particles with a corresponding location (*X*, *Y*, and *Z*), momentum (*dX*, *dY*, and *dZ*) and energy (*E*) [5]. Subsequent simulations can use this PhSs as the starting point for MC simulations, reducing the resource requirements. However, in medical physics, particle numbers in the order of magnitude of $10^{8}$ or higher are required to simulate dose distributions or images with reasonable uncertainty. Thus, the generation of PhSs is time consuming and requires the storage of large data files. In addition, dedicated PhSs are required to cover changes in the X-ray production, such as changing tube voltage or filters. Hence, overall storage capacity requirements are in the order of magnitude of hundreds gigabytes.

An alternative to the use of PhSs are analytical descriptions of the X-ray source properties [6]. This is an efficient but cumbersome approach, typically optimised for individual geometries and use cases. [7] proposed the use of neuronal networks, specifically Generative Adversarial Network (GAN) to reduce the required modelling effort while still benefiting from a semi-analytical source model [7]. This goes alongside with currently increased developments of using neural networks for medical image processing [8], [9], [10], [11].

The application of GAN is a straightforward decision, as this kind of neural network synthetically generates samples of a learned distribution [12]. The resulting models also considerably reduce disk storage space.

However, this implies that data are generated for a specific configuration, necessitating the training of numerous models for multiple energies and filter settings.

In this study, we demonstrated how full MC modelling of an X-ray imaging system can be accomplished. The model was experimentally validated and the practical utility enhanced by a conditional GAN, which allows efficient modelling of multiple X-ray energies. Finally, a comparison between the traditional PhSs and the conditional GAN was performed based on a generated X-ray image.

## 2 Material and methods

**Fig. 1:**
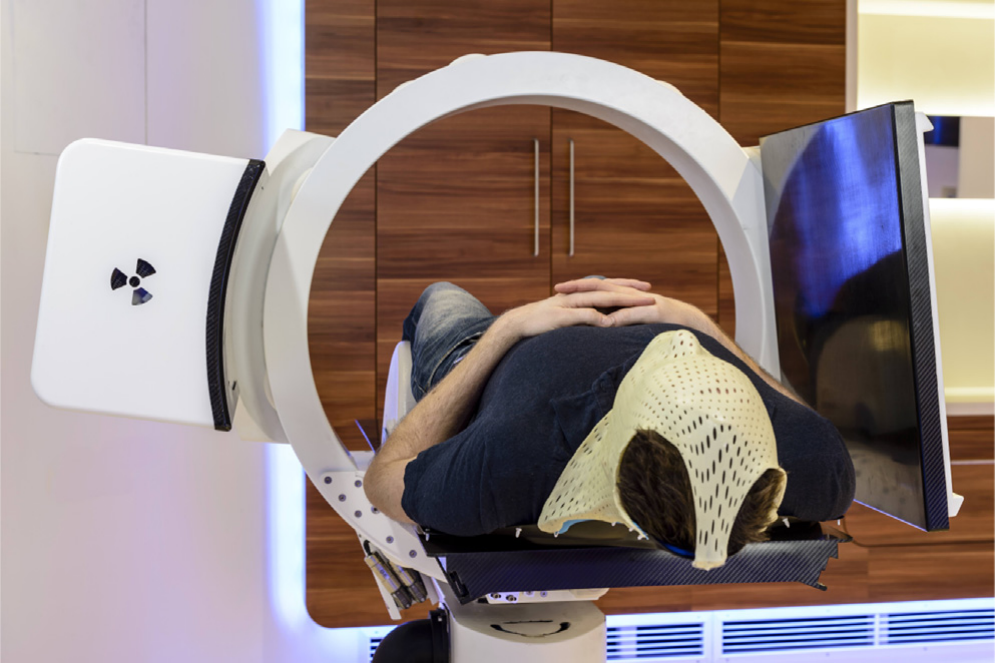
ImagingRing system as installed in a clinical treatment room at MedAustron ion therapy facility, depicting X-ray source and detector mounted on the ring. [(c) Kästenbauer/Ett].

The X-ray based in-room imaging system (${\mathrm{ImagingRing}}^{\mathrm{TM}}$, medPhoton GmbH, Salzburg, Austria) installed at the MedAustron ion therapy center [13], [3], [14] was investigated in this study (see Fig. 1). The main parts of the ImagingRing system are a 2D-collimated X-ray monoblock system and a solid-state amorphous silicon detector, both mounted on a ring. The system allows longitudinal movement of the ring and independent rotation of the X-ray source and detector. Four independent movable jaws are mounted in the X-ray source. A primary collimator and a flattening filter made of Al are attached to the mono block system.

Modelling followed an approach recently published by van der Heyden et al. [3, Fig. 1].

From the various X-ray energies that can be produced, four are employed in clinical protocols at MedAustron, *i.e.* 60, 80, 100, and 120 keV. Measurements were performed with the clinically used energies with tube currents of 20 mA, if not specified otherwise.

The half-value layer (HVL), the focal spot, and the Heel effect were measured and henceforth compared to the MC model of the X-ray source.

### 2.1 Experimental ImagingRing characterisation

The photon energies were characterised by HVLs in aluminium, measured with a NOMEX® multimeter detector (PTW, Freiburg, Germany)^2^. To obtain data for reconstructing the photon energy spectra, multiple measurements were performed with additional filtration material positioned in front of the detector. In detail HVLs were measured for four different filter settings, *i.e.* no filter, 3 mm Al, 0.5 mm Cu, and 3 mm Al plus 0.5 mm Cu.

The horizontal and vertical profiles of the focal spot of the X-ray source were measured with a PTW slit camera (PTW, Freiburg, Germany), as described in [3]. The slit camera was placed as close as possible to the exit window (*i.e.* 0.8 cm) and aligned with the primary central axis of the X-ray tube. The enlargement factor was 7.27. The focal spot was measured for different tube voltages, currents, and exposure times. The ImagingRing system provides two focal spot sizes, depending on the tube current. In this study only the smaller focal spot was investigated. The dimension of the focal spot projection was defined at the full width at 15% of the maximum intensity [3].

To quantify the Heel effect all filters, including the flattening filter, were removed from the X-ray head. The measurements were performed with an independent detector system (Lynx, IBA dosimetry, Schwarzenbruck, Germany) which was aligned perpendicular to the center of the X-ray beam. Measurements were performed for 120 kV using one pulse of 10 ms.

### 2.2 ImagingRing Modelling with Monte Carlo

The open-source MC toolkit GATE-RTion v 1.0 [15], [16], a clinically validated version based on GATE v8.1 [17], [18], alongside Geant4 v10.3 patch 03 was used for all MC simulations. Simulations employed the low energy electromagnetic physics list Penelope as only electromagnetic interactions were necessary. Modelling accuracy of the underlying physics models depends on the target-material, energy and emission angle and is usually within 10–30% [19]. An overview of the parameters employed for simulations can be found in Table 1. Data evaluation was performed using in-house developed python and R scripts. A detailed drawing of the components of the ImagingRing X-ray source can be found in van der Heyden et al. [3, Fig. 1].

**Table 1:** Parameters employed for MC simulations.

Parameter		Value
Physics list		empenelope
Production and tracking cuts	air	1 mm
	anode, flattening filter	0.001 mm
	detector	0.01 mm
Variance reduction	$e^{-}$ Bremsstrahlung splitting	1000
	$e^{-}$ ionisation splitting	1000

To correctly take into account all effects occurring inside the X-ray head, such as self-filtration of the anode (heel effect), a full MC modelling of the X-ray head was performed, starting with the electron beam impinging on the anode. Based on computer-aided design (CAD) drawings and tube specifications provided by the manufacturer, all geometries and materials were modelled inside GATE. To improve performance, variance reduction techniques were implemented. In the anode, physical processes leading to photon emission were split, *e.g.* the final state of the process was repeated multiple times. A splitting factor of 1000 was chosen for maximum efficiency. The number of initial particles simulated was adjusted to achieve a statistical uncertainty below 0.1%. The parameters were optimised for four measured voltage settings, *i.e.* 60 kV, 80 kV, 100 kV, and 120 kV.

In a first step, monoenergetic electron beams impinging the anode were simulated ranging from 30–120 keV. The X-ray beam exiting the head was focused on an aluminium block, providing depth dose curves and consequently HVL values. In a second step, the electron energies were superimposed and their respective weighting optimised to best match the measured HVL values. This was repeated for all modelled kV settings (*i.e.* 60, 80, 100, 120 kV). The resulting electron energy spectra were then used as input for all further simulations.

The focal spot profiles were approximated in the simulation by the superposition of Gaussian spots resulting in four Gaussian-shaped electron beams for the focal spot.

To verify the Heel effect, a simulation was performed without the primary collimator, flattening filter, and secondary collimators (E = 120 keV), which mimics the experimental setup with the most pronounced Heel effect.

### 2.3 Machine learning based phase space modelling

The above mentioned MC model was used to generate $10^{8}$ particles per energy. The data were split into two portions (50/50) for training and validation. As a baseline the GAN model of Sarrut et al. was re-implemented for single energy PhS generation [7]. The model translates random latent space inputs into a five dimensional tensor giving spacial coordinates (*X* and *Y*) as well as the momentum (*dX*, *dY*, and *dZ*) of a single particle. This is referred to as traditional GAN.

In addition, a conditional GAN model was constructed to increase the efficiency in the aspect of multiple source energies (*e.g.* 60, 80, 100, and 120 keV). For this purpose an additional embedding layer was introduced which uses the label of the source energy and selects a four dimensional vector which is concatenated to the six dimensional latent vector. Spectral normalisation [20] for the discriminator and the non-saturated loss function with R1 regularisation was applied [21], [22].

Both, generator and discriminator, have three hidden layers consisting of 400 neurons each and use ReLU as activation function except for the last hidden layer of the generator utilising a sigmoid activation function. The models were trained for 20 epochs with a learning rate of $2\times 10^{-5}$ and the RMSProp optimiser. One epoch represents one run overall available training data. A batch size of $10^{4}$ was used. For all experiments, these settings were identical.

The generation performance was further improved by including the exponential moving average (EMA) of the generator model [21], [22]. In a hyperparameter search, two different loss functions (Wasserstein loss and non-saturated loss) were compared, with/without spectral normalisation, and with/without EMA to validate the improved performance. Spectral normalisation was only applied to the discriminator.

It is difficult to quantify the performance of a generative model as the samples generated should only mimic the real samples. To track the performance of the model, a histogram loss was included which compared absolute difference in terms of counts of $10^{6}$ particles of the validation data set to randomly sampled particles of the model. The hyper-parameter search was then performed to decrease the sum of the single parameter histogram losses over the generated vectors:

(1) $$L_{\mathrm{total}}=L_{\mathrm{hist}}^{X}+L_{\mathrm{hist}}^{Y}+L_{\mathrm{hist}}^{\mathrm{dX}}+L_{\mathrm{hist}}^{\mathrm{dY}}+L_{\mathrm{hist}}^{\mathrm{dZ}}+L_{\mathrm{hist}}^{\mathrm{energy}}$$

with $L_{\mathrm{hist}}$ is defined as:

(2) $$L_{\mathrm{hist}}=\overset{n}{\underset{i=0}{\sum }}|{\mathrm{counts}}_{\mathrm{cGAN}}^{i}-{\mathrm{counts}}_{\mathrm{PhS}}^{i}|$$

where *n* is the number of bins and *counts* is the number of particles for either PhS or GAN generated data of the $i^{\mathrm{th}}$ bin. The total loss for conditional models was calculated by summing all histogram losses of each trained energy and divide it by the total number of energies.

Moreover, we explored the latent space by interpolating between different energies to describe the possibility to generate energies that were not part of the training process. Therefore, we applied a linear interpolation between the two nearest existing embedding tensors, after the first neural network layer which can be expressed as:

(3) $$p=G_{2-3}(\lambda G_{1}(x,E(y_{1}))+(1-\lambda )G_{1}(x,E(y_{2})))$$

where $G_{x}$ is the corresponding generator depth, $E(y_{x})$ the embedding tensors of the given label and λ is the interpolation coefficient.

Quantitative results are obtained by interpolating between two known energies (*e.g.* 60 and 100 keV to generate 80 keV) with λ set to 0.5. Differences E are given as percentage disagreements between real and synthetically generated counts which can be seen as:

(4) $$E=\frac{L_{\mathrm{hist}}}{n_{\mathrm{parameters}}\times n_{\mathrm{particles}}}\times 100$$

where $n_{\mathrm{parameters}}$ is the number of generated parameters (n = 6) and $n_{\mathrm{particles}}$ is the number of generated particles for the validation (n=$10^{6}$).

For the final validation, the model was implemented in C++ to use the particles generated directly in GATE. This enabled the simulation of an X-ray image acquired from a CT object. For qualitative assessment, both, the traditional PhS, as well as the conditional GAN were used as a photon source, respectively, providing $1\times 10^{11}$ primary photons to simulate an image at an X-ray energy of 100 keV. The X-ray image (see Fig. 6) was simulated with completely open secondary collimators, providing the maximum field size. A variance reduction technique, a so-called track-length estimator [23], was employed to reduce noise at the detector.

## 3 Results

### 3.1 Comparison between measurements and MC simulation

A comparison of the measured and MC simulated HVL values of the ImagingRing system is shown in Table 2. Maximum deviations were (0.9 ± 0.2) mm for the thickest filter setting and 100 kV.

**Table 2:** Comparison of measured and simulated HVL values in mm aluminium of the ImagingRing system.

	Filters	60 kV	80 kV	100 kV	120 kV
Measured HVL in Al [mm]	no filter	3.0 ± 0.2	4.0 ± 0.2	4.7 ± 0.2	5.6 ± 0.2
	3 mm Al	3.6 ± 0.2	4.9 ± 0.2	5.6 ± 0.2	6.7 ± 0.2
	0.5 mm Cu	5.5 ± 0.2	7.4 ± 0.2	8.6 ± 0.2	9.8 ± 0.2
	3 mm Al + 0.5 mm Cu	5.7 ± 0.2	7.6 ± 0.2	8.9 ± 0.2	10.0 ± 0.2
					
Simulated HVL in Al [mm]	no filter	3.4	4.3	4.9	5.9
	3 mm Al	3.6	4.7	5.3	6.4
	0.5 mm Cu	5.7	7.7	8.9	10.1
	3 mm Al + 0.5 mm Cu	5.7	6.9	8.0	9.4

The uncertainties of the HVL measurements include type A and type B uncertainties according to [24], where the type B uncertainty of the NOMEX® multimeter is the dominating factor.

**Fig. 2:**
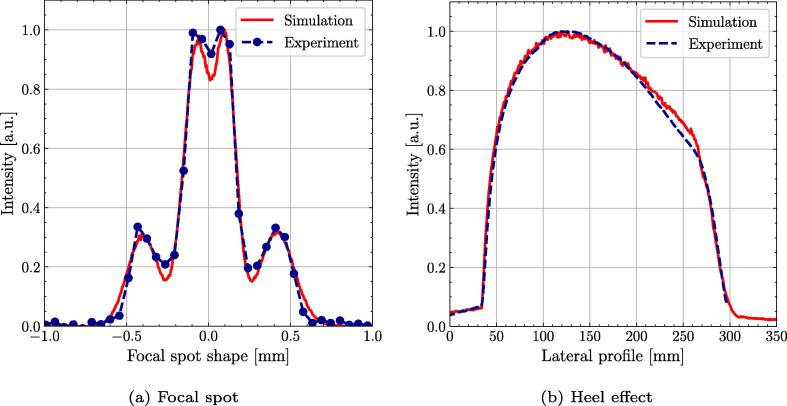
Comparison of the MC modelled X-ray source with measured data for the horizontal focal spot shape profile at 100 kV measured using the slit camera (a) and the lateral intensity profile without flattening filter, showing the heel effect at 120 kV (b).

The focal spot was found to be Gaussian-shaped in a vertical plane, while in the horizontal plane a more complex shape was identified, where a superposition of 4 Gaussian shapes was found to be adequate for MC modelling (Fig. 2a). The dimension of the focal spot was measured with (0.60 ± 0.03)mm and (0.70 ± 0.04)mm in a vertical plane and horizontal plane, respectively. The uncertainty of the focal spot was mainly driven by positioning of the slit camera and variations of the X-ray source. To quantify the latter we measured the focal spot in regular intervals over one year.

A comparison of the horizontal intensity profile measured to the Lynx detector to MC modelling of the X-ray head with all beam shaping elements removed, prominently showed the heel effect (Fig. 2b). Both modalities agree well, with deviations less than 10% on the right fall-off of the profile (Fig. 2b).

### 3.2 Machine learning based phase space modelling

**Fig. 3:**
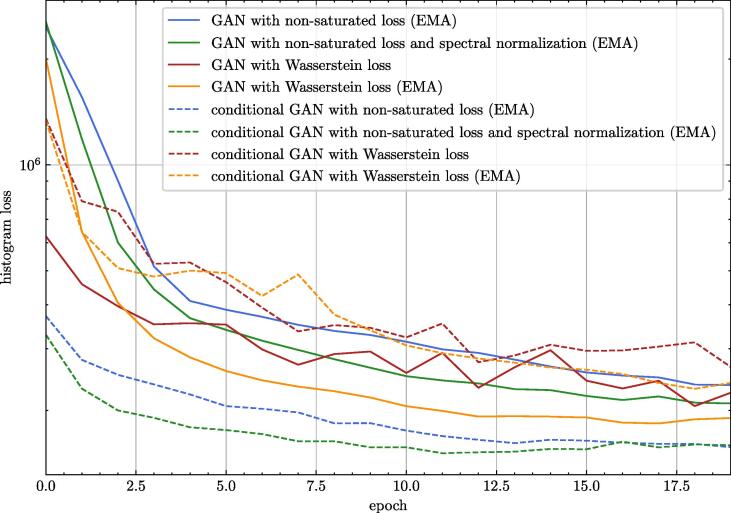
Validation loss comparison between different settings for 100 kV. EMA is the exponential moving average of the model parameters. One epoch represents one run over all available training data.

**Fig. 4:**
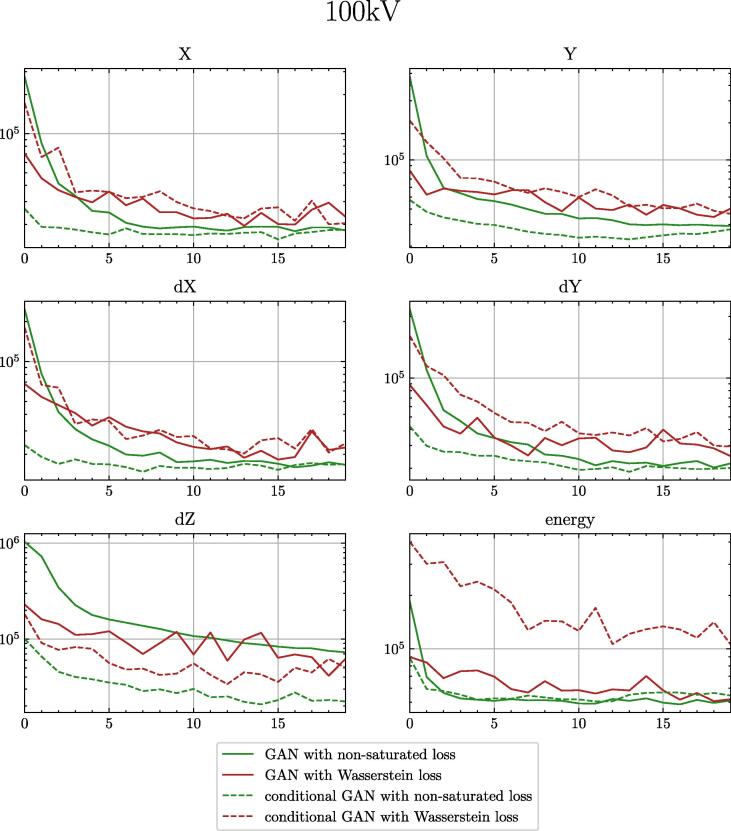
Histogram loss for the single generated parameters of the 100 kV PhS data. Direct comparison of the conditional GAN and the traditional GAN. Including the starting setup (with Wasserstein loss) and the final model (with non-saturated loss).

**Fig. 5:**
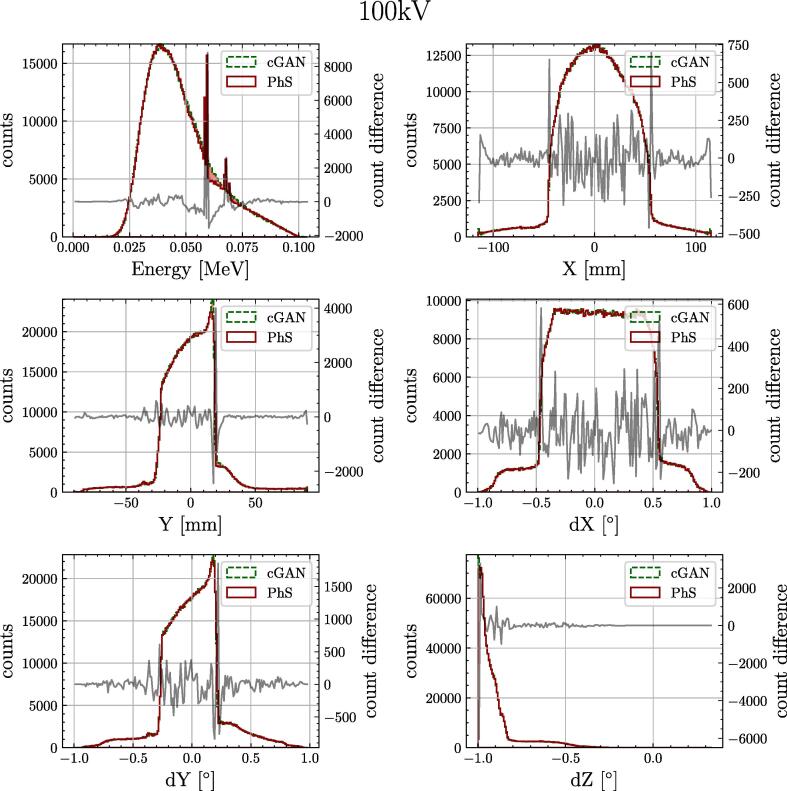
Comparison of real and synthetically generated PhS spectra of the single parameters. On the left y-axis the number of counts and on the right y-axis the count difference between MC and conditional GAN data is displayed. The histograms are computed from $10^{6}$ particles.

The improvement of the model measured with a histogram loss can be seen in Fig. 3. The influence of changing different parameters on the histogram loss is given in Table 3. The largest improvement was recognised when the non-saturated loss function with R1 regularisation was used instead of the Wasserstein loss. The changes in the single parameters of the PhS data can be seen in Fig. 4. A comparison of the final spectra for 100 kV can be found in Fig. 5.

**Table 3:** Validation histogram loss for the different settings for conditional GAN and traditional GAN. The columns ”norm” and ”EMA” indicate if spectral normalization and exponential moving average of the weights were applied. Bold numbers give better results for one specific setting (per row). Values marked with an asterisk indicate the overall best result of all experiments.

parameters			conditional GAN				traditional GAN			
loss	norm	EMA	60 kV	80 kV	100 kV	120 kV	60 kV	80 kV	100 kV	120 kV

wasserstein	FALSE	FALSE	4.87	4.07	4.44	4.38	**3.95**	**3.75**	**4.21**	**4.26**
wasserstein	FALSE	TRUE	**3.99**	3.66	3.99	3.83	4.29	**3.49**	**2.74**	**3.35**
non-saturated	FALSE	TRUE	**2.64**∗	**2.39**∗	**2.62**	**3.04**	4.38	3.94	3.07	3.31
non-saturated	TRUE	TRUE	**2.85**	**2.48**	2.65	3.15	3.38	3.17	**2.58**∗	**2.86**∗

Interpolating after the first hidden layer achieved better results than directly interpolating the embedding layer vectors. Smooth interpolations are possible from different energies even from those that were not part of the training data set. The latent space interpolation achieved 2.78% and 3.48% while the corresponding embedded energies (80 kV and 100 kV) achieved 2.52% and 2.55% Spectra comparisons are available in the supplementary material. As we can only provide quantitative results for two energies, we added an interpolation animation as a qualitative result in the supplementary material.

**Fig. 6:**
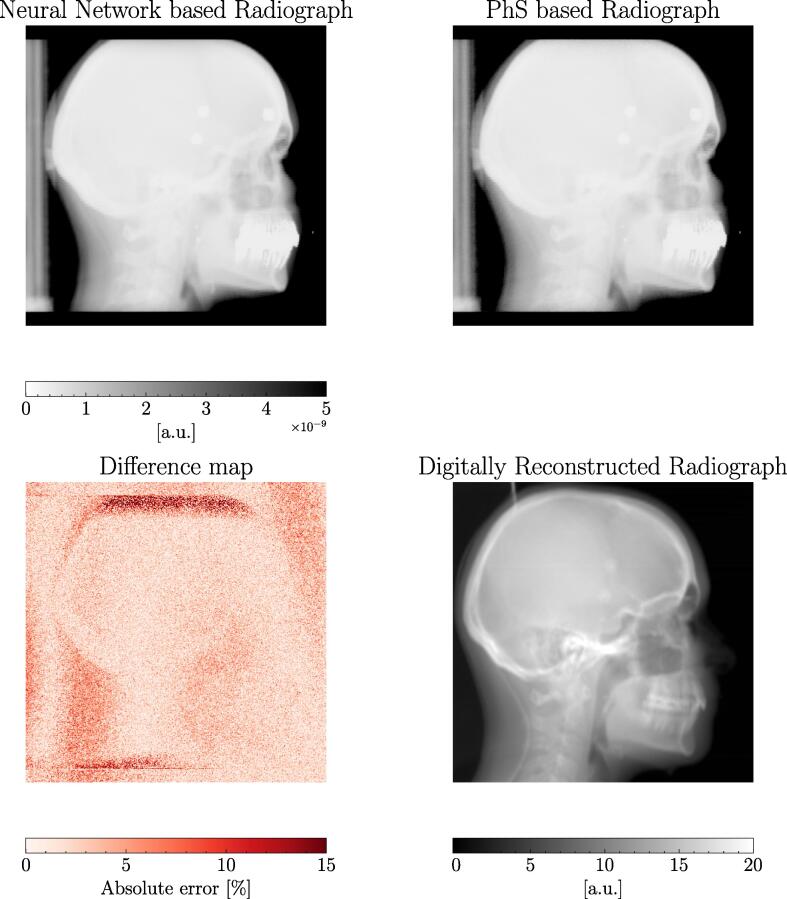
Simulated X-ray with PhS file (right upper graph) and conditional GAN (left upper graph) in comparison to the digitally reconstructed radiograph (right lower graph). The difference map (left lower graph) represents the relative error between the PhS and GAN simulation.

The output of the 100 kV X-ray image of the original PhS and the conditional GAN can be seen in Fig. 6. The qualitative comparison of the difference map showed good agreement. Relevant deviations are present in soft tissue regions as it can be seen in the throat area (*i.e.* up to 6%). Larger differences within the patient contour are caused by a limitation of the maximum field of view of the modelled imaging system.

## 4 Discussion

Creating a full MC model of an X-ray system, starting from the initial electron beam is a complex task, that requires detailed information about the system and extensive calculation resources. One incident electron created on average between $7\times 10^{-3}$ and $1.2\times 10^{-1}$ photons, depending on the initial energy. Only a small fraction of these photons continue towards the exit window, which considerably reduces the efficiency of the simulation. While very inefficient, to our knowledge, this is the best way to simulate all processes inside the X-ray head, such as the self-filtration and partial beam hardening inside the anode, non-homogeneous photon spectra etc. However, the calculation efficiency makes its use prohibitive for routine simulations.

The hyper-parameter search showed that the conditional GAN and the traditional GAN gave with different settings better results in terms of the histogram metric. For both types of generative model, better results were observed using a non-saturated loss function with R1 regularisation. The EMA did not always improve the results by much but helped making the synthesis more reliable and consistent. Applying the spectral normalisation for the discriminator helped especially the traditional GAN setting and led to a slight degradation of the conditional GAN (see Table 3). Largest errors were present in the region of the characteristic peaks, especially for 100 and 120 kV. The sub-optimal modelling of the characteristic peaks will limit the image quality if these qualities contribute dominantly, such as soft-tissue imaging for breast cancer screening. However, for most X-ray imaging applications the contribution to image quality of the characteristic peaks is limited. Conditioning the model onto multiple different energies made it possible to interpolate between different energies enabling to generate energy spectra which were not part of the training. Interpolating between the first hidden layer transformations of two energies (*e.g.* 60 and 100 kV) made it possible to compare it with data which was available. The interpolation resulted in similar histogram metrics in comparison to the embedded energies of the conditional GAN (2.78% vs 2.52% for 80 kV and 3.48% vs 2.55% for 100 kV). This can further be used to reduce the number of energies simulated via MC which are required for the model training.

An additional advantage over PhS files is that for training only a small fraction of the amount of photons compared to a typical X-ray simulation was required, further reducing the amount of calculation resources.

The conditional GAN can be called via a C++ implementation of the generated torch script which can be then applied instead of the PhS file as it was also performed by Sarrut et al. [7]. The generation of photons was about 2.4% slower than compared to reading the PhS files. However, this time difference is marginal and still saved in this scenario 22 GB of disk space. The final X-ray simulation can be seen in Fig. 6 where the difference map showed low differences in the patient object projection.

## 5 Conclusion

A good match between measurement and simulation data for HVL, focal spot size and heel effect could be shown using the full MC modelling of the ImagingRing X-ray head. A novel conditional GAN was developed and trained on four different X-ray energies, enabling an efficient method to use a complex source model with limited storage and calculation overhead. A considerably lower number of particles were required to train the conditional GAN compared to a traditional PhS approach, substantially reducing calculation resources for modelling. Furthermore, this model showed good results when interpolating between different energy embeddings, allowing to synthesise energies that were not part of the training. This can further reduce the number of required MC simulations for single X-ray devices in the future, considerably increasing modelling and calculation efficiency.

## Declaration of Competing Interest

The authors declare that they have no known competing financial interests or personal relationships that could have appeared to influence the work reported in this paper.

## Footnotes

2 www.ptwdosimetry.com, December 2021
