# Supplementary material for: Efficient full Monte Carlo modelling and multi-energy generative model development of an advanced X-ray device
Source: Z Med Phys. 2022 Jun 7;33(2):135–45. doi: 10.1016/j.zemedi.2022.04.006 (PMC10311273; doi:10.1016/j.zemedi.2022.04.006)
Supplement: Supplementary file 1 [file mmc1.pdf]

Supplementary material

Training plots

In figure 1, 2 and 3 the equivalent loss plots can be found as for the 100 keV) plot in the main manuscript (cf. Figure 3). All energies showed the best results with the conditional GAN, trained with the non-saturated loss function. In figure 4, 5 and 6 a clear improvement can be seen for all parameters if the non-saturated loss function is implemented instead of the Wasserstein loss.

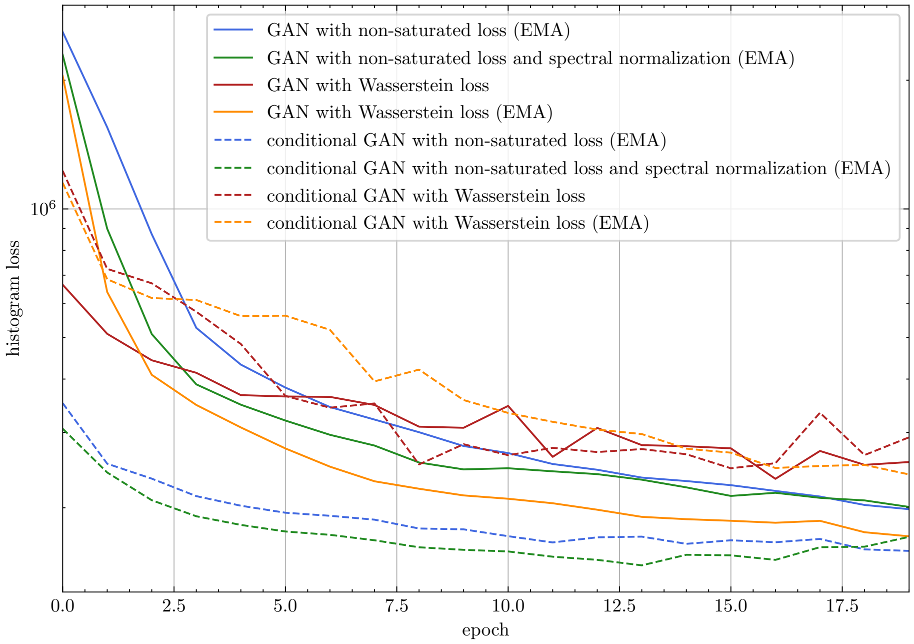

Figure 1: Comparison of real and synthetically generated PhS spectra of the single parameters of 60 keV. On the left y-axis the number of counts and of the right y-axis the count difference between MC and conditional GAN data is displayed. The histograms are computed from 10^6 particles.

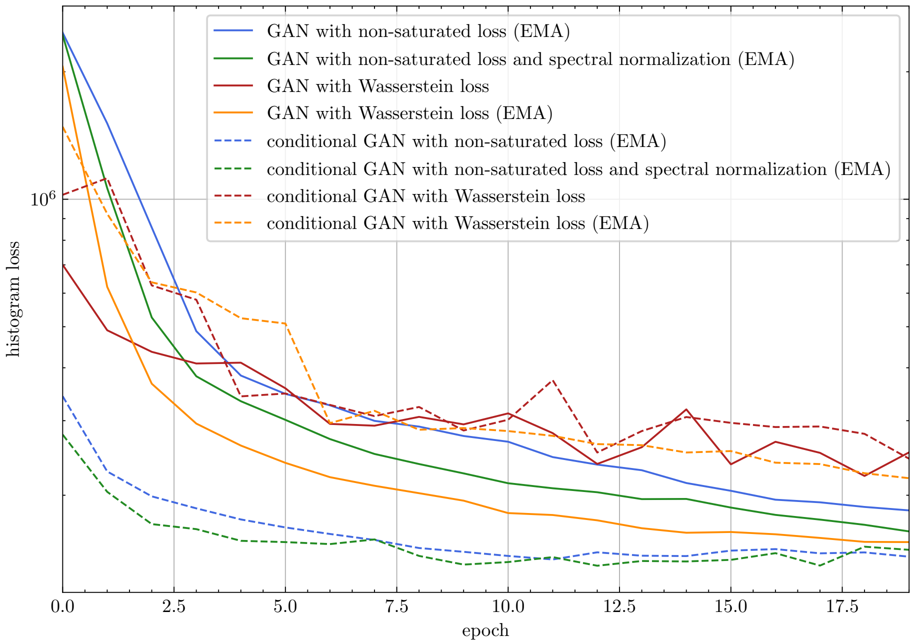

Figure 2: Comparison of real and synthetically generated PhS spectra of the single parameters of 80 keV. On the left y-axis the number of counts and of the right y-axis the count difference between MC and conditional GAN data is displayed. The histograms are computed from 10^6 particles.

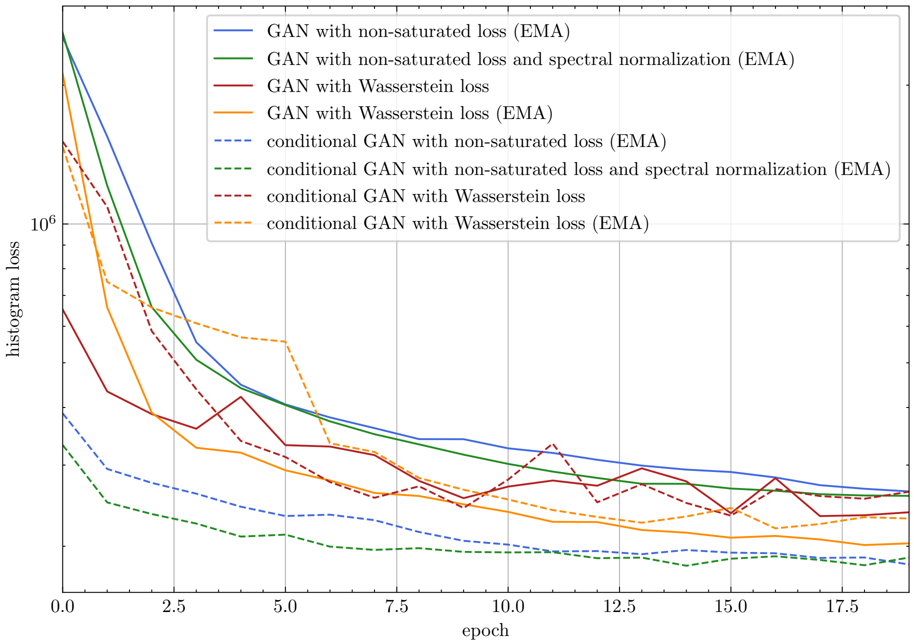

Figure 3: Comparison of real and synthetically generated PhS spectra of the single parameters of 120 keV. On the left y-axis the number of counts and of the right y-axis the count difference between MC and conditional GAN data is displayed. The histograms are computed from 10^6 particles.

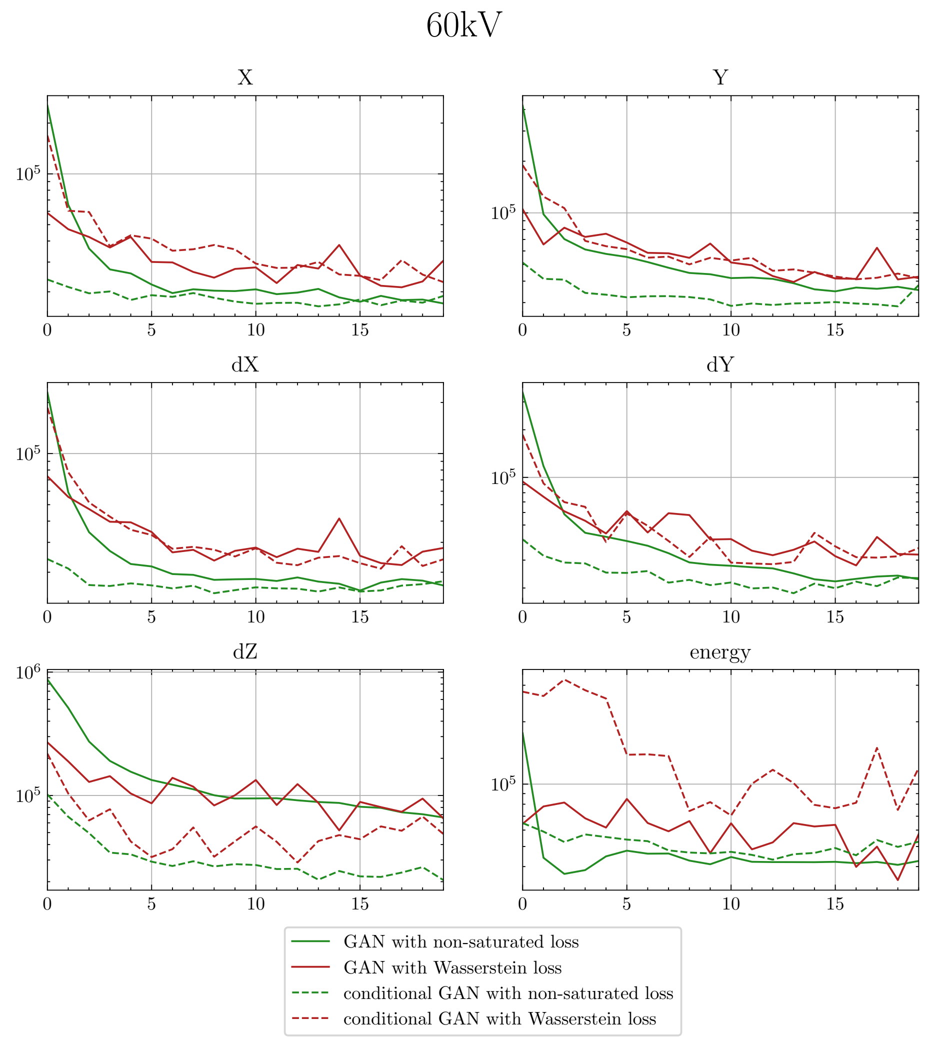

Figure 4: Comparison of real and synthetically generated PhS spectra of the single parameters of 60 kV. On the left y-axis the number of counts and of the right y-axis the count difference between MC and conditional GAN data is displayed. The histograms are computed from 10^6 particles.

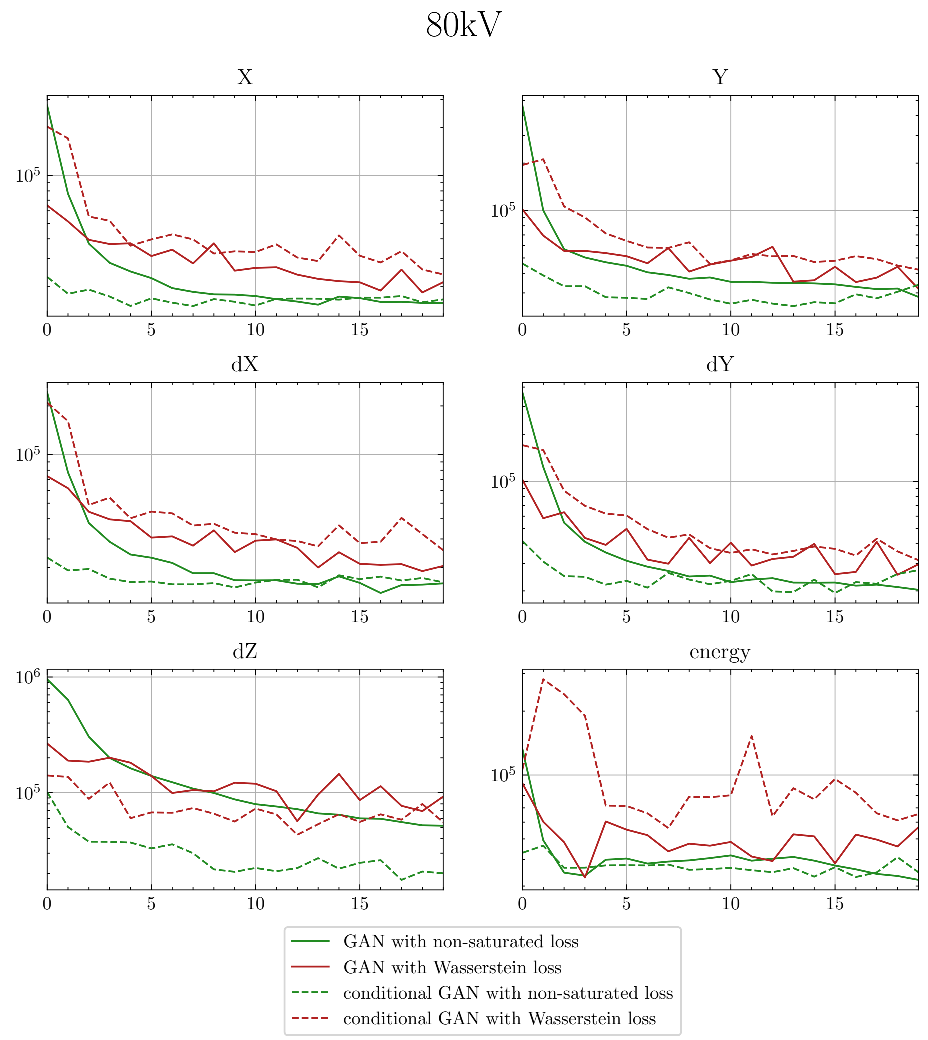

Figure 5: Comparison of real and synthetically generated PhS spectra of the single parameters of 80 kV. On the left y-axis the number of counts and of the right y-axis the count difference between MC and conditional GAN data is displayed. The histograms are computed from 10^6 particles.

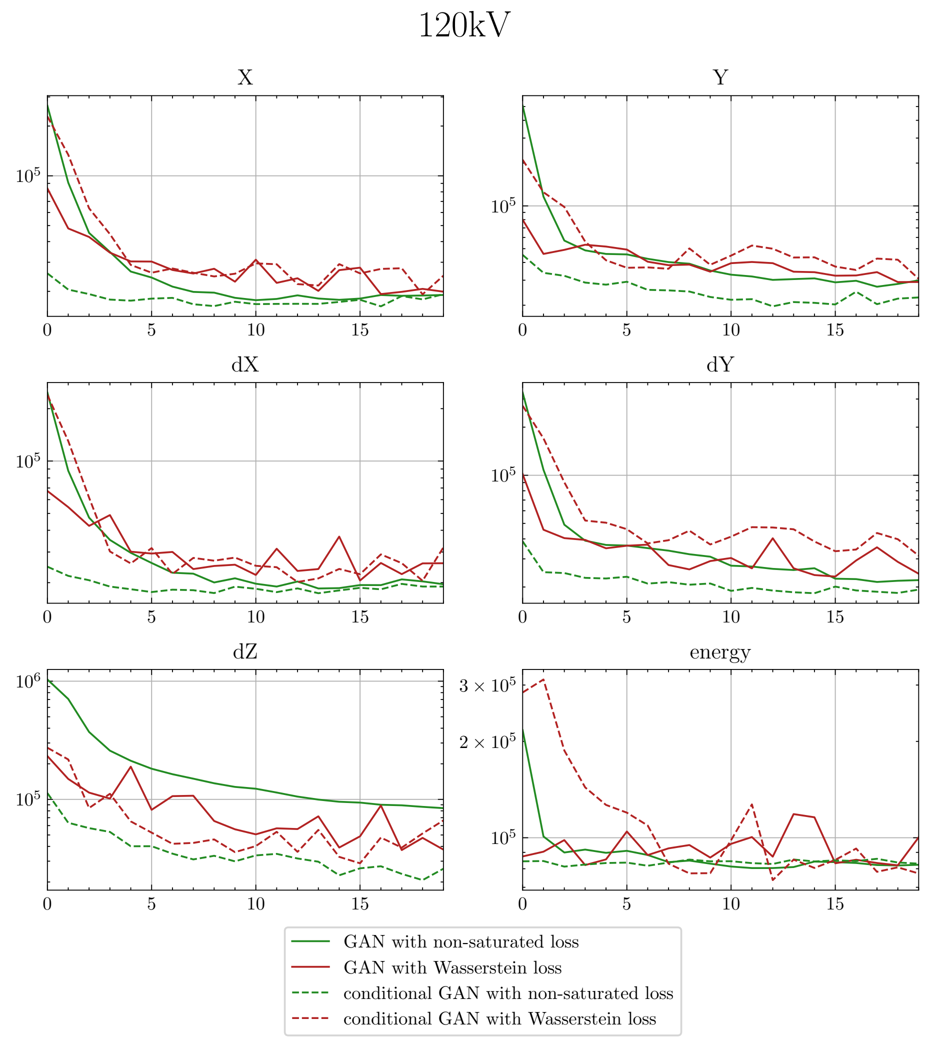

Figure 6: Comparison of real and synthetically generated PhS spectra of the single parameters of 120 kV. On the left y-axis the number of counts and of the right y-axis the count difference between MC and conditional GAN data is displayed. The histograms are computed from 10^6 particles.

Conditional Generative Adversarial Network results

In Figure 7, 8 and 9 the spectral histograms of the phase space can be seen.

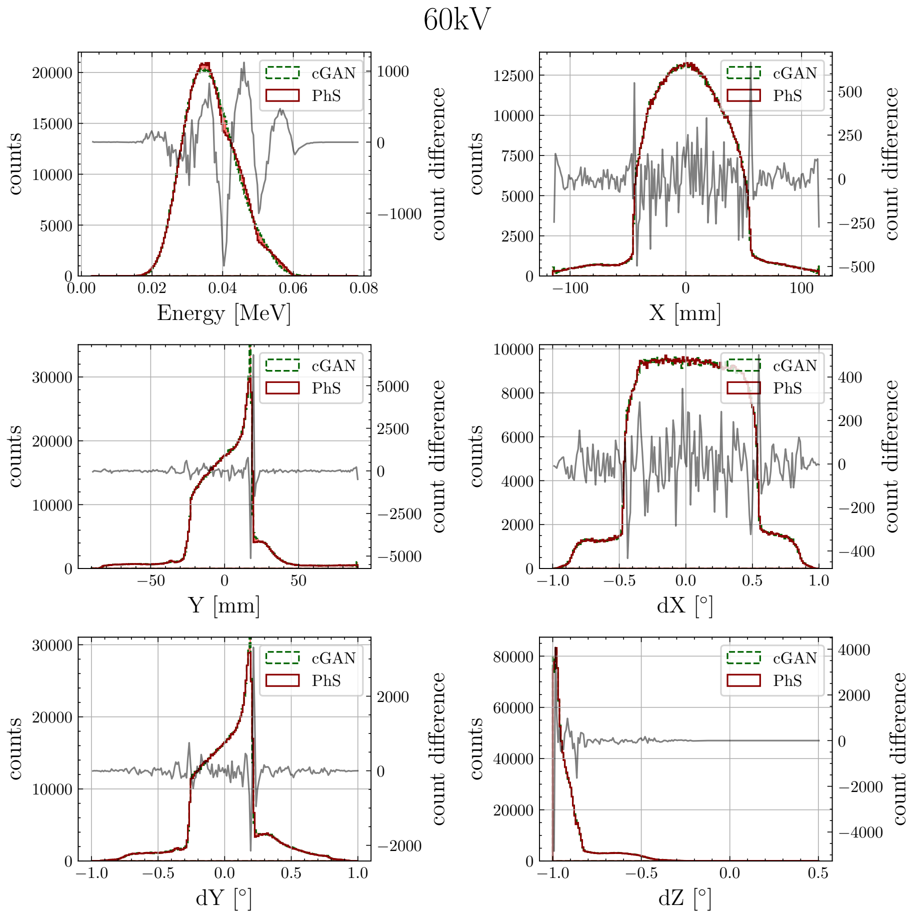

Figure 7: Comparison of real and synthetically generated PhS spectra of the single parameters of 60 kV. On the left y-axis the number of counts and of the right y-axis the count difference between MC and conditional GAN data is displayed. The histograms are computed from 10^6 particles.

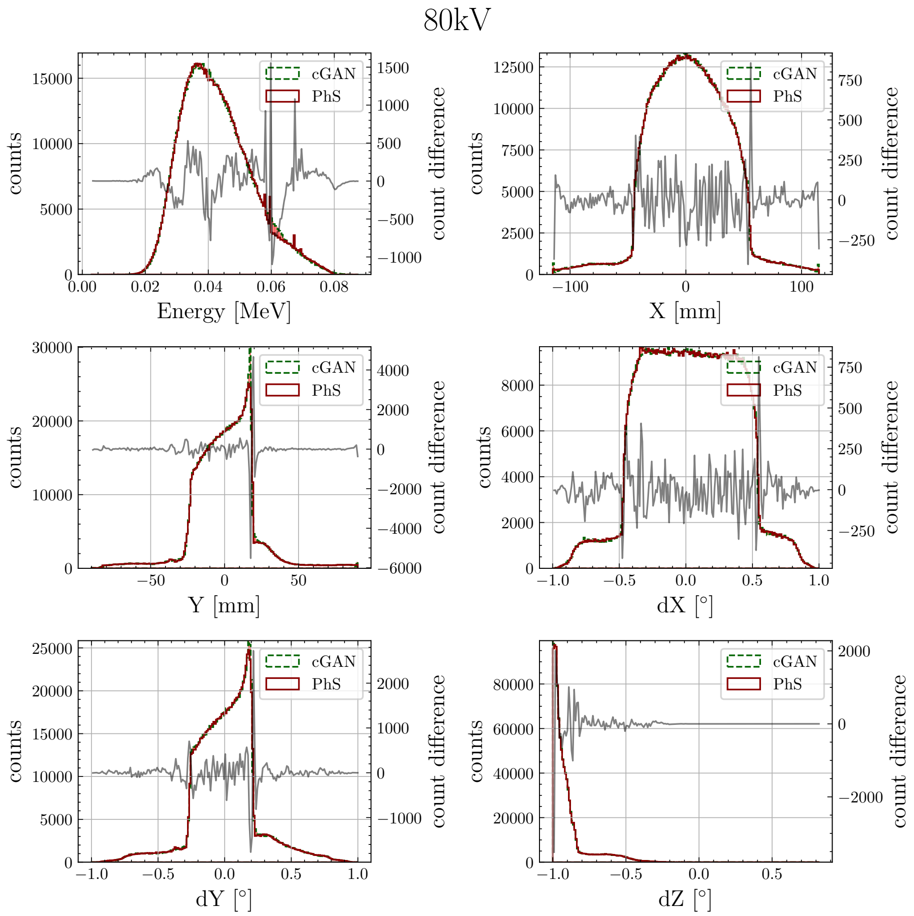

Figure 8: Comparison of real and synthetically generated PhS spectra of the single parameters of 80 kV. On the left y-axis the number of counts and of the right y-axis the count difference between MC and conditional GAN data is displayed. The histograms are computed from 10^6 particles.

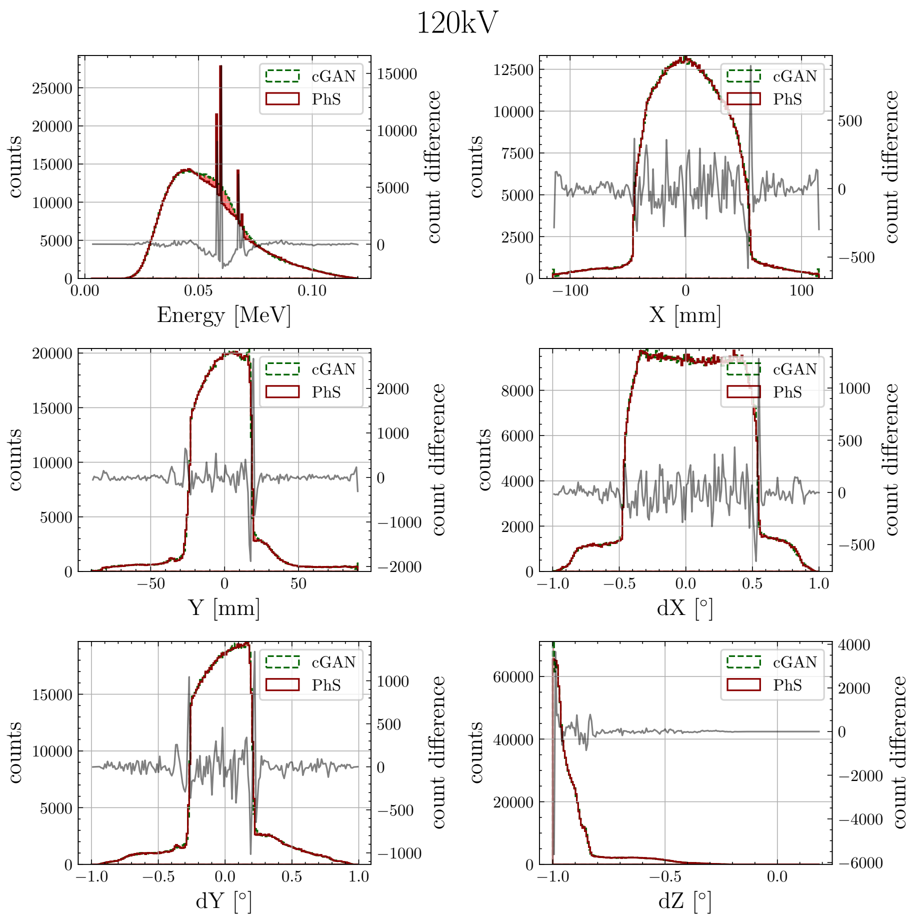

Figure 9: Comparison of real and synthetically generated PhS spectra of the single parameters of 120 kV. On the left y-axis the number of counts and of the right y-axis the count difference between MC and conditional GAN data is displayed. The histograms are computed from 10^6 particles.

Interpolation results

In Figure 10 and 11, the spectra of the interpolation method can be seen. Note that the results have larger differences as the interpolation is performed in between two embedding vectors 40 keV apart. Interpolation can be seen in the wandb report and were performed between the two closest embedded energies (*i.e.* 60 keV and 80 keV).

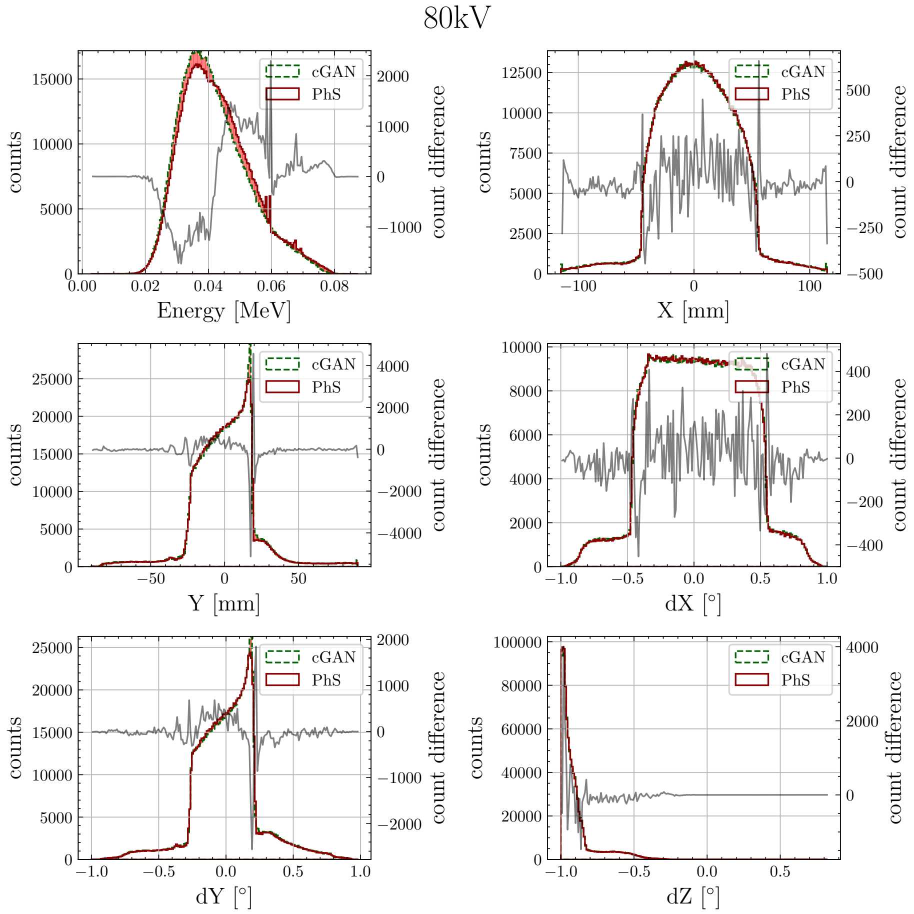

Figure 10: Comparison of real and synthetically generated PhS spectra of the single parameters of 80 keV for the interpolated energy. On the left y-axis the number of counts and of the right y-axis the count difference between MC and conditional GAN data is displayed. The histograms are computed from 10^6 particles.

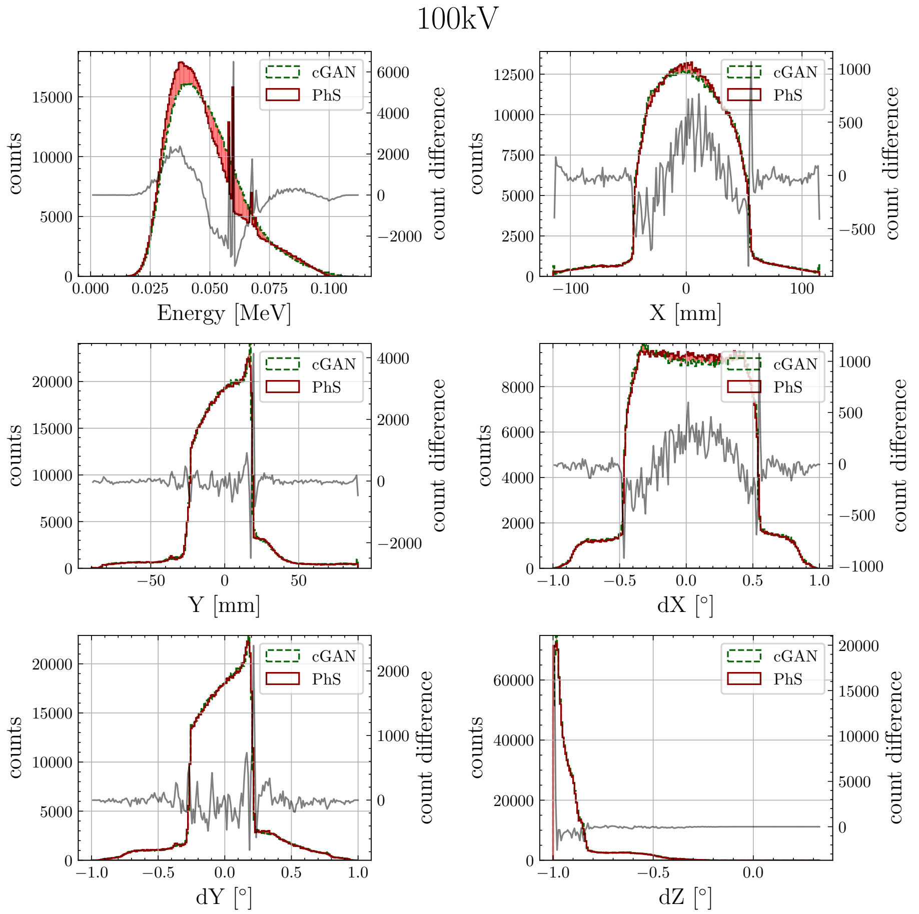

Figure 11: Comparison of real and synthetically generated PhS spectra of the single parameters of 100 keV for the interpolated energy. On the left y-axis the number of counts and of the right y-axis the count difference between MC and conditional GAN data is displayed. The histograms are computed from 10^6 particles.
